# The potential of convolutional neural networks for identifying neural states based on electrophysiological signals: experiments on synthetic and real patient data

**DOI:** 10.3389/fnhum.2023.1134599

**Published:** 2023-06-02

**Authors:** Fernando Rodriguez, Shenghong He, Huiling Tan

**Affiliations:** MRC Brain Network Dynamics Unit, Nuffield Department of Clinical Neurosciences, University of Oxford, Oxford, United Kingdom

**Keywords:** neural decoding, adaptive deep brain stimulation, brain-computer interface, convolutional neural networks, local field potentials, neural oscillations, signal processing, electrophysiology

## Abstract

Processing incoming neural oscillatory signals in real-time and decoding from them relevant behavioral or pathological states is often required for adaptive Deep Brain Stimulation (aDBS) and other brain-computer interface (BCI) applications. Most current approaches rely on first extracting a set of predefined features, such as the power in canonical frequency bands or various time-domain features, and then training machine learning systems that use those predefined features as inputs and infer what the underlying brain state is at each given time point. However, whether this algorithmic approach is best suited to extract all available information contained within the neural waveforms remains an open question. Here, we aim to explore different algorithmic approaches in terms of their potential to yield improvements in decoding performance based on neural activity such as measured through local field potentials (LFPs) recordings or electroencephalography (EEG). In particular, we aim to explore the potential of end-to-end convolutional neural networks, and compare this approach with other machine learning methods that are based on extracting predefined feature sets. To this end, we implement and train a number of machine learning models, based either on manually constructed features or, in the case of deep learning-based models, on features directly learnt from the data. We benchmark these models on the task of identifying neural states using simulated data, which incorporates waveform features previously linked to physiological and pathological functions. We then assess the performance of these models in decoding movements based on local field potentials recorded from the motor thalamus of patients with essential tremor. Our findings, derived from both simulated and real patient data, suggest that end-to-end deep learning-based methods may surpass feature-based approaches, particularly when the relevant patterns within the waveform data are either unknown, difficult to quantify, or when there may be, from the point of view of the predefined feature extraction pipeline, unidentified features that could contribute to decoding performance. The methodologies proposed in this study might hold potential for application in adaptive deep brain stimulation (aDBS) and other brain-computer interface systems.

## 1. Introduction

### 1.1. Algorithmic approaches for decoding information from neural activity

**FIGURE 1:**
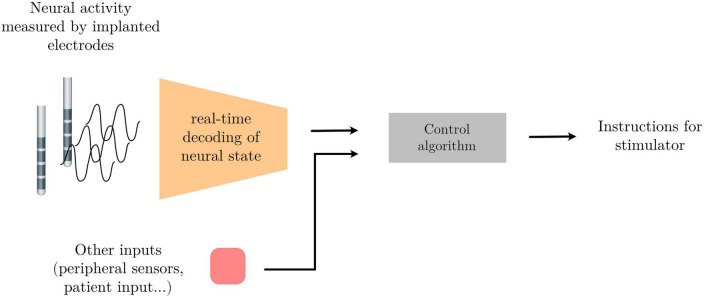
Schema of a signal processing pipeline commonly needed for adaptive deep brain stimulation (aDBS) or brain computer interface (BCI) applications.

Neural activity, as measured through electrophysiological recordings, consists of multi-channeled timeseries data containing oscillatory patterns that reflect an underlying circuit state. This brain state might in turn be indicative of different neural or mental states, the detection of which can be used to drive external devices to build an alternative communication pathway between the brain and the external world; or indicative of pathological states that could be altered using electrical stimulation for therapeutic gain. Finding the patterns within the neural activity that best differentiate neural states from one another, and developing systems that recognize these patterns in real-time can be used as part of a broader control system to achieve these ends. One example of such a system is adaptive Deep Brain Simulation (aDBS) – a therapeutic approach in which the brain stimulation parameters are automatically adapted to the settings most appropriate for an identified brain state (see Figure 1 below).

In practice, this neural decoding capability is achieved by training machine learning systems that infer these brain states in real-time. These models rely on quantifying the presence of relevant activity patterns within the neural signals, with these patterns often taking the form of synchronous bursts of oscillatory activity at canonical frequencies with specific waveform characteristics (Neumann et al., 2022). It thus becomes crucial to find the methods that are best suited to extract relevant patterns from the neural activity and quantify them as features. Within a paradigm where only discrete neural states are present, algorithmic approaches that have been used in the previous literature can be classified into one of two types, with the methodological differences described below:

#### 1.1.1. The classical machine learning, or feature-based approach

When using this approach, the information of interest (termed *features*) that is considered to contain information related to the relevant neural states, is manually extracted from the raw waveforms through filtering, smoothing, and other numerical techniques. This often relies on spectral power estimation methods based on periodograms and the fast Fourier transform (Ahn et al., 2020; He et al., 2021; Bijanzadeh et al., 2022) or on digital filtering (Shah et al., 2018). Features in the time-domain have also been shown to contribute to decoding (Shah et al., 2018). In addition, efforts are underway to expand the feature set to encompass further waveform characteristics such as the waveform sharpness, waveform asymmetry, or cross-frequency coupling.^1^ Once a set of predefined features has been extracted from the raw waveforms, it is fed into a classifier that, by modeling the statistical relationships between features and labels in the training data, learns a function to map combinations of waveform-derived features onto the probability of a corresponding neural state being present. At inference time, if the relationships between features and underlying neural states learned during training are meaningful, the classifier can predict what neural state is most likely to be present given the features extracted from the incoming neural waveforms. When using this approach, significant consideration must be given to feature design, since these features are the basis upon which the classifier is able to learn relevant statistical relationships. This approach has been extensively used for current movement state-driven aDBS implementations (He et al., 2021; Merk et al., 2022b). Within this framework, some methods derived from deep learning can be incorporated. In Golshan et al. (2020), the neural signals were first decomposed into 2-dimensional time-frequency images using the wavelet transform, and then a convolutional neural network was applied on the 2D images for decoding.

#### 1.1.2. The end-to-end deep learning approach

Differing from the feature-based paradigm, in which raw waveforms are first transformed into a feature set that is subsequently fed into a classifier, within the end-to-end deep learning approach, the feature extraction pipeline (from raw waveforms to feature space) is not manually designed but is rather the result of an optimization procedure. The features are extracted by numerical operations that take place within the network and are discovered during the optimization process. To process naturalistic signals such as images or waveforms, the numerical operation of choice is oftentimes a convolution. This operation is well suited to identify activity taking place at specific frequencies, since the convolution theorem dictates that a convolution in the time-domain is equivalent to a product in the frequency domain: 𝔉 {*f* * *g*} = 𝔉 {*f*} ⋅ 𝔉 {*g*} with 𝔉 denoting the Fourier transform and ⋅ the convolution operation. This approach has not only yielded improvements over the feature-based approach in computer vision (image processing) and speech processing (where signals also take the form of waveforms), but has also been successfully applied to a number of electrophysiology-related applications both within and outside of the field of DBS (Golshan et al., 2020; Gao et al., 2021; Peterson et al., 2021; Shoeibi et al., 2021; Supakar et al., 2022). The wavelet transform, which is one of the methods that is used most often for time-frequency decomposition of electrophysiological signals to quantify the presence of activity at specified frequencies in a time-resolved manner, is a parameterised convolution. We therefore propose that a convolutional neural network (CNN) can be thought of as a numerical operation capable of extracting relevant features from waveforms in both the frequency and time domain (e.g., quantifying the presence of transient activity in relevant frequency bands). Within this end-to-end framework, the CNNs take the raw waveforms as inputs, requiring minimal preprocessing. The networks are parametrized with random values at the beginning, and an optimal value for these parameters is then iteratively found by gradually minimizing a loss objective through gradient descent. With this approach, it is no longer necessary to manually design and implement a feature extraction pipeline (the function that maps raw oscillatory timeseries into a feature vector), since these processing steps are learnt from the data directly. This can result in more optimized processing pipelines by avoiding the reliance on manual tuning required when implementing one’s own feature extraction procedure (e.g., setting the boundaries that delimit frequency bands of interest, or manually tuning the numerical methods used to identify harmonic patterns). Training deep learning systems, especially end-to-end CNNs, however, comes with its own set of challenges. Firstly, special attention must be paid to make sure that the architecture (i.e., the parametrized operations) is well-suited to solve the task at hand. Secondly, in order for the CNN to be trained properly and for the training process to appropriately converge to a point that generalizes well to previously unseen inference-time data (avoid under and overfitting), networks have strict requirements about the statistical distribution of their input data and depend on the quantity and also the quality of data available at training time.

### 1.2. Signal processing for aDBS

Adaptive deep brain stimulation (aDBS), a technique by which the stimulation parameters or intensity of delivered through DBS electrodes can be adapted according to some feedback signal related to brain state or pathology, shows promise for increasing the efficacy, reducing side effects, and expanding the therapeutic window of DBS for a range of movement disorders (Guidetti et al., 2021). Feedback signals used in aDBS can be extracted directly from neural activity, for instance the local field potentials measured through DBS electrode contacts, or neurophysiological signals measured from additional electrodes placed in other areas of the brain.

Local field potentials measured through DBS electrode contacts can be used to decode movement or other pathological states using machine learning methods, so that stimulation is only delivered when necessary and overall current delivery is minimized. It has been shown that this kind of aDBS can maintain equivalent therapeutic efficacy compared to conventional DBS while minimizing side effects affecting speech, gait or postural stability (Little et al., 2016; Little and Brown, 2020; He et al., 2021). Furthermore, aDBS might also safeguard patients against developing habituation or tolerance from the therapeutic effects of stimulation over time (Peters and Tisch, 2021).

Most current work in the field of aDBS relies on the feature-based approach, i.e., on manually extracting a set of predefined features from waveforms, and training classifiers to learn the statistical relationships between the manually extracted features and the probability of a corresponding neural state being present. Some work uses what can be regarded as a combination of both approaches, i.e., instead of training a deep learning system that operates on the raw waveform directly, a deep learning system is trained to operate on processed features or derivative representations of the raw timeseries data, such as a time-frequency decomposition (see Golshan et al., 2020; Thenaisie et al., 2022).

Here, we aim to explore the feasibility and compare the performance of these two approaches for training real-time compatible decoding systems based on LFP waveforms, and to document advantages and drawbacks of each depending on the characteristics of the oscillations. In particular, we aim to explore the potential of end-to-end CNN for decoding based on neural activities in the context of aDBS, where the signal-to-noise ratio can be low and variable from patient to patient, and the quantity of data available for training is limited. We motivate the use of deep learning approaches by pointing to successful applications of neural networks in other areas of electrophysiology. In Sun et al. (2022), the authors were able to leverage the representational power of deep neural networks to enable high-fidelity spatiotemporal source reconstruction of high-density EEGs, all the while reducing the need to manually tune solvers. In our case, we postulate that when using the feature-based approach, relevant information about neural states contained within the LFP waveforms could be unintentionally discarded during the filtering and feature extraction process, yielding a poorer decoding performance. To test this hypothesis, we first designed an end-to-end CNN structure capable of extracting various features from the neural signals that can be recorded during a short experimental session with a patient. We then generated synthetic waveforms with different activity patterns and trained both feature-based classifiers and deep learning-based classifiers to distinguish between simulated states. By simulating data and thus being able to control the input signals’ features and signal-to-noise ratios, we were able to probe the ability of different systems to model specific patterns of activity. We then trained these same systems on real patient LFP waveforms recorded from externalized essential tremor patients for the task of distinguishing movement from rest and compared their decoding performance.

The remainder of this study is structured as follows: first we explain how we generate synthetic data to mimic oscillatory patterns that one might find in electrophysiological recordings. We then implement different feature-based and deep learning-based machine learning systems and apply them to the task of decoding states using both synthetic and real patient data. We then benchmark these systems against one another, document respective benefits and drawbacks and conclude this study. The methods developed here can be applied to both local field potential (LFP) activity measured from DBS electrodes as well as to other types of electrophysiological data including electrocorticography (ECoG), which has also been explored within the context of aDBS (Merk et al., 2022b).

## 2. Materials and methods

### 2.1. Dataset I: synthetic data

In order to benchmark the ability of different algorithmic approaches to extract information from waveforms containing various oscillatory patterns, we generate synthetic datasets that aim to mimic some of the patterns found in neural time-series data that are modulated by different brain states. The generated datasets have a total length of 20 min, of which the first 80% (16 min) are used as training data, and the last 20% (4 min) as validation data. The sampling rate of the synthetic signals is 2048 Hz.

In the synthetic data, neural states are modeled as two-state, first-order Markov process, with each state having a minimum duration of 5 s and the probability of transitioning from that state being randomly drawn from 𝒰[0.3, 0.4], leading to average state duration of 10.92 +/− 7.94 s. This ensures a degree of stochasticity in the duration of states but ensures that the states are present in the dataset in similar proportions, avoiding imbalances. We choose this timing because it mimics the in-clinic setting in which decoding systems are conventionally trained in the context of DBS, with patients being asked to perform trials of movement followed by rest. This was the data recording paradigm used in He et al. (2021). Each state is characterized by relative differences in the presence of one of the waveform patterns (e.g., higher activity vs. lower activity in a specific band) that have been previously shown to be modulated by movements or pathology in signals recorded from movement disorder patients. We generate a total of 7 oscillatory patterns, including the presence of oscillations at specific frequencies, the non-linearity of waveforms, oscillatory activity modulated by the phase of another oscillator at a different carrier frequency (phase-amplitude coupling), the duration of transient bursts of synchronized activity, and phase differences between channels. The patterns are generated within a single channel except for the cross-channel PAC case, and for the cross-channel phase shift case, both of which require two channels. The task we pose to the classification systems is to distinguish between the two states.

1/f-shaped pink noise (generated using the *colorednoise*^2^ python package) is added to the signals at different intensities to modulate the noise content, where the signal-to-noise ratio is defined as


$$S\,N\,R=\frac{r\,m\,s\,\left(s\,i\,g\,n\,a\,l\right)}{r\,m\,s\,\left(n\,o\,i\,s\,e\right)}$$

with *rms* being the root-mean-square, or energy, of the signal. For each oscillatory pattern, we perform SNR sweeps ranging from 0.2 (5× the noise power relative to signal power) to 2.0 (twice more signal power than noise power) at 0.05 increments. For each of these noise levels (36 in total), we generate 5 datasets. This results in a total of 180 datasets.

The oscillatory patterns that characterize the different states are:

#### 2.1.1. Power changes in the beta and gamma bands

**FIGURE 2:**
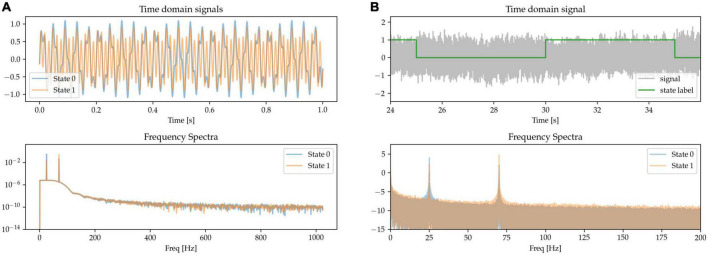
The presence of oscillators at 25 Hz (beta oscillation) and 70 Hz (gamma oscillation) characterizes two simulated states. **(A)** Time-series excerpts of the two states without any additive 1/f-noise (top), and their corresponding frequency spectra (bottom). **(B)** The two states, with added noise, are simulated as a Markov process (top). As shown in the frequency spectrum, the two states are distinguished by the beta and gamma peaks (bottom).

Modulation in the power of the canonical beta (approx. 13 – 30 Hz) and gamma (approx. 40 – 90 Hz) frequency bands has been widely reported during movements in the cortical-subcortical motor network. Here, we synthetically mimic this pattern by generating states containing oscillations of different amplitudes at these canonical frequencies and summing them together. The resulting frequency spectrum is characterized by the presence of sharp peaks at each of the relevant frequencies above the noise floor, but with different amplitudes for the two frequencies characterizing the two states. State 0 is characterized by higher power in beta band and lower power in the gamma band when compared with State 1 (see Figure 2, where in the frequency spectra the relative peak heights indicate oscillatory power differences).

#### 2.1.2. Beta waveform sharpness

**FIGURE 3:**
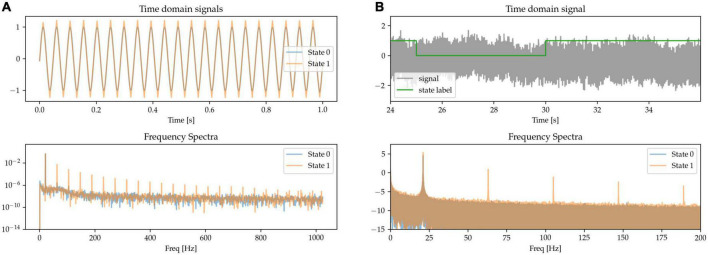
**(A,B)** Sinusoidal (State 0) and sharp (State 1) waveforms distinguish the two states, with matched power at the base oscillatory frequency. The sharp waveforms have odd harmonics in the frequency spectrum, contrasting with the pure sinusoid which has a single peak at the oscillatory frequency (bottom).

The sharpness of the beta waveform has been documented as a marker of circuit pathology in Parkinson’s disease, in particular due to it being modulated by beta burst dynamics (Cole et al., 2017; Yeh et al., 2020). A sharp beta waveform can be generated as a non-sinusoidal, triangular waveform at beta frequency (see State 1 in Figure 3A), in comparison to a sinusoidal waveform (State 0). Power at the base beta frequency is matched for both states. When expressing this sharp triangular waveform in the frequency domain using a basis of pure sinusoids (such as is the case with the Fourier transform), the symmetry between the positive and negative phases of the oscillation (sharpness at both the peak and trough of the waveform) results in the even components of the harmonics canceling each other out. The frequency spectrum is thus characterized by the presence of odd harmonics, as shown for State 1 in the frequency spectra of Figure 3, whereas there is only one single peak at the base frequency when the waveform is a pure sinusoid (State 0).

#### 2.1.3. Non-linear phase

**FIGURE 4:**
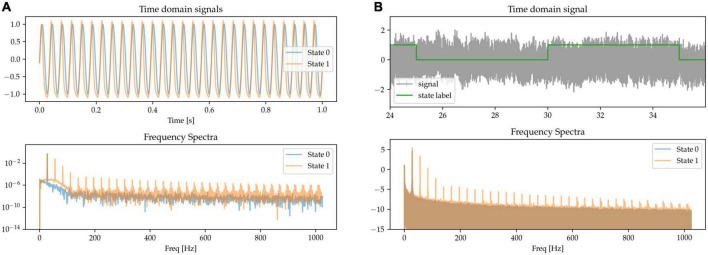
**(A,B)** The states are characterized by their degree of phase non-linearity, i.e., approximately sinusoidal (State 0), or with sharp peaks and wide troughs (State 1). In the frequency spectrum, this non-linearity is represented in the form of harmonics.

Besides waveform sharpness, other kinds of waveform non-linearities (deviations from sinusoidality) have been reported as relevant features of neural oscillations (Quinn et al., 2021). Here, we implement oscillations with non-linear phase as having a variable degree of asymmetry between the durations of the first and second half of the oscillatory cycle. This leads to fast trough-to-peak (rising) and slow peak-to-trough (falling) times, resulting in waveforms with narrow peaks and wide troughs, as shown in the waveform data of State 1 in Figure 4. The asymmetry between the positive and negative phase of the oscillation results in the presence of both odd and even harmonics in the Fourier-derived frequency spectrum. The signals are scaled to equate for power in the base beta frequency.

#### 2.1.4. Theta-gamma phase amplitude coupling (PAC)

**FIGURE 5:**
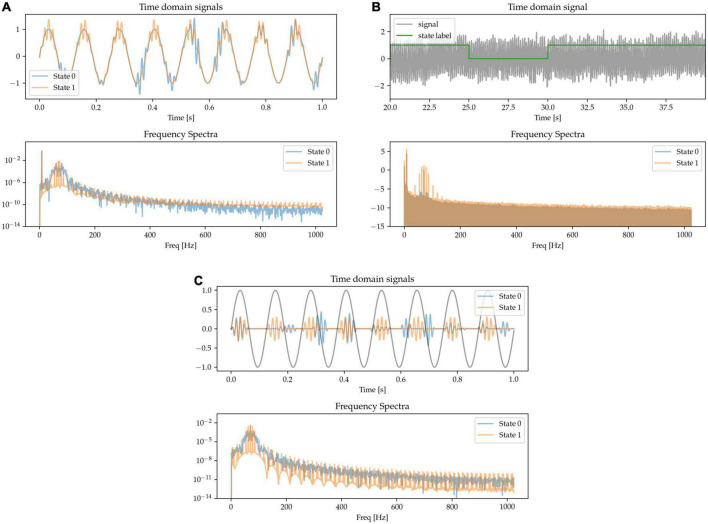
Gamma appears at random times (State 0), or phase-coupled to a lower-frequency oscillation (State 1) either in the same channel **(A,B)** or a different one **(C)**.

There is evidence of neural oscillations at different frequencies interacting with each other, with special interest being directed toward patterns in which the phase of lower frequency oscillations at a carrier frequency modulate the power of higher frequency activity (Tort et al., 2010). For example, theta-gamma phase amplitude coupling in the hippocampus has been shown to support long-term spatial memory retrieval (Vivekananda et al., 2021) and theta-gamma phase amplitude coupling has also observed been in other brain areas during different physiological functions (Canolty et al., 2006; Lizarazu et al., 2019). Increased beta-gamma phase amplitude coupling in the motor cortex is also indicative of pathological activity in Parkinson’s disease (Meidahl et al., 2019). Here, we simulate this waveform feature by generating transient bursts of gamma activity that are either coupled to the positive phase of an underlying theta oscillation (State 0) or appear at random times (State 1). Overall gamma power is adjusted to be matched for the two states. The gamma activity is either coupled to a theta signal in the same channel (Figure 5A) or in a different one (cross-channel PAC, Figure 5C). The frequency spectrum is characterized by harmonic microstructure around the gamma peak (Figure 5 frequency spectra).

#### 2.1.5. Cross-channel phase shift

**FIGURE 6:**
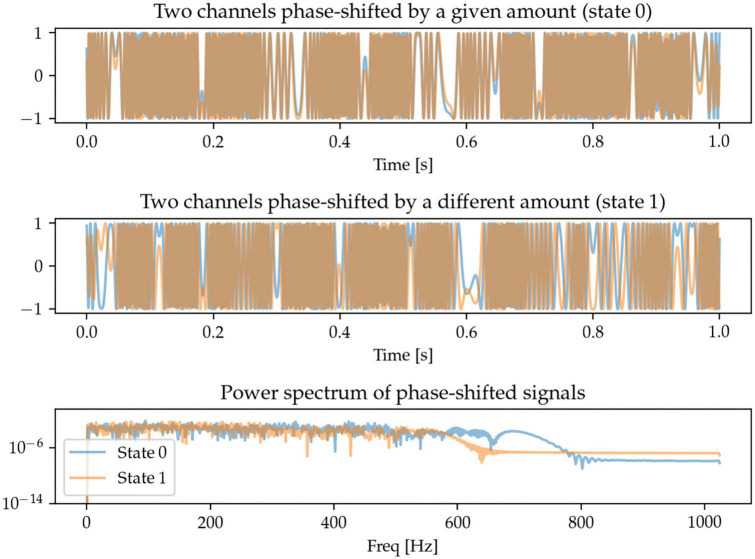
Each state contains two randomly generated signals that are phase-shifted, with the defining feature being the degree of phase-shift. Since the signals are random, so is their power spectrum, and there is no distinguishable spectral pattern between the two states.

One potentially interesting approach to characterize oscillations across brain regions (and across measurement sensors) is to be able to quantify the extent to which the same oscillations take place in different regions, but with a time-delay (or phase-shift) between them. Here, we generate signals in two sensor space channels that are phase-shifted by different amounts. The frequency content of the signals is random and there is thus no identifiable pattern in the power spectrum. The task for the machine learning models is to be able to distinguish between these random signals with two different degrees of phase-shift (Figure 6).

#### 2.1.6. Beta burst length

**FIGURE 7:**
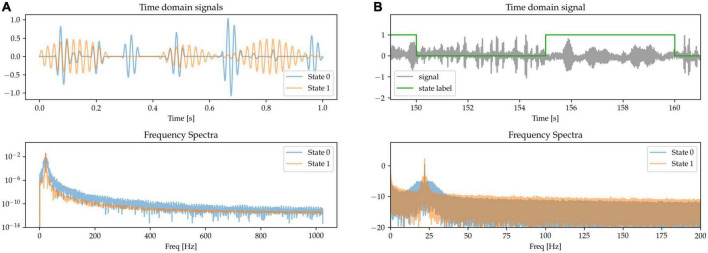
**(A,B)** State 0 is characterized by short beta bursts, and State 1 by long bursts. Waveform amplitudes are adjusted to match average power at the peak beta frequency.

The role of the temporal dynamics of beta burst in basal ganglia LFPs has been identified as a relevant marker of circuit state and of pathological activity, especially in terms of the length of the bursts (Tinkhauser et al., 2018). To model this, we generate randomly occurring bursts of beta activity, but with different average lengths for each of the two different states: in State 0, episodes of increased beta power (beta bursts) tend to be very brief in duration; whereas in State 1, the beta bursts are longer. We adjust the amplitude of the bursts to equalize beta power at the peak frequency (Figure 7).

### 2.2. Dataset II: LFP-based movement decoding

In order to benchmark the ability of different systems to decode states not only from synthetic data but also from real patient LFPs, we make use of an existing dataset consisting of LFPs recorded from the bilateral ventralis intermediate nucleus of the thalamus (Vim-thalamus) of essential tremor (ET) patients externalized after lead implantation surgery while they perform three self-paced upper-limb movement tasks. This dataset was first published in He et al. (2021).

The dataset comprises 8 patients during a pegboard insertion game, a posture holding task with arms stretched out in front of them, and a rice pouring task (pouring rice back and forth between two cups). Patients were instructed to perform the movement for approx. 30 s, and then to rest for another 30 s, repeating this 6–10 times. This was repeated both with bilateral 130 Hz DBS stimulation on and off at a single preselected stimulating contact. This results in 8.4 ± 1.8 min (mean ± std. deviation) of recording for each task for each participant. Recordings were made 4 or 5 days after electrode implantation with externalized leads and included bipolar EMG measurements of the flexor carpi radialis (both arms) as well as triaxial accelerometer measurements (accelerometers taped to the dorsum of each hand). The signals were sampled at 2048 Hz using a TMSi Porti amplifier (TMS International, the Netherlands).

As a pre-processing step, signals were highpass-filtered (4th order butterworth filter, forward mode) with a cut-off frequency of 1 Hz. Movement labels were extracted from either the accelerometer or EMG signals by thresholding the RMS power of the signals of interest. All available LFP channels across both hemispheres are used as input into the classifiers.

Due to the short duration of the datasets (6–10 trials in each), we use a 5-fold cross-validation strategy to benchmark the classifiers, i.e., each dataset is split into 5-folds of equal length and at any one time, four of them are treated as training data with the fifth being treated as validation data. In total, the data consists of 200-folds: 8 patients × 3 tasks (some tasks were not recorded for certain patients) × 2 stimulation conditions. The training set duration is 6.7 ± 1.9 min (mean ± std. deviation), and the validation set duration is 1.7 ± 0.5 min (mean ± std. deviation).

### 2.3. Decoding systems

We classify the decoding systems used in this work into one of two categories: feature-based and deep learning-based. Both make use of data to learn the mapping function:


f:w⁢a⁢v⁢e⁢f⁢o⁢r⁢m⁢s→p⁢(s⁢t⁢a⁢t⁢e|w⁢a⁢v⁢e⁢f⁢o⁢r⁢m⁢s),

The two approaches, however, achieve this in different ways, with the feature-based approach requiring one to first derive a feature vector manually from the waveforms, and subsequently using classical machine learning techniques to estimate a mapping function from feature space to the probability of a state being present.


$$f_{f\,e\,a\,t\,u\,r\,e\,s}:f\,e\,a\,t\,u\,r\,e\,s\to p\,\left(s\,t\,a\,t\,e|f\,e\,a\,t\,u\,r\,e\,s\right)$$

By contrast, the deep learning approach learns a direct, end-to-end mapping from the waveforms to the state of interest by directly optimizing the parameter set θ


$$f_{D\,L}:w\,a\,v\,e\,f\,o\,r\,m\,s,\theta \to p\,\left(s\,t\,a\,t\,e|w\,a\,v\,e\,f\,o\,r\,m\,s,\theta \right).$$

We implement two feature-based models [implemented using python’s scikit-learn package (Pedregosa et al., 2011)] and three deep learning-based models [implemented using PyTorch’s python API (Paszke et al., 2019)]. For all these systems, waveforms are split into 500 ms moving window epochs with 100 ms overlap (one output every 400 ms, or at 2.5 Hz).

#### 2.3.1. Feature-based model I: spectral SVM

**FIGURE 8:**
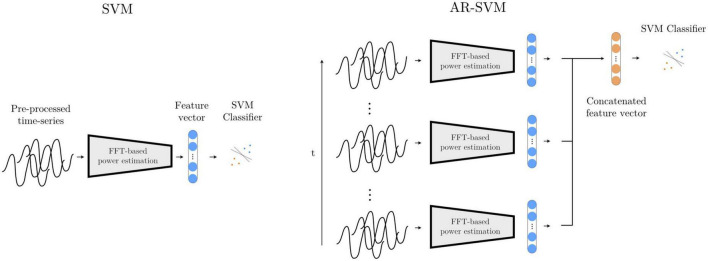
Feature-based models SVM and AR-SVM. In the SVM approach **(left)**, pre-processed waveforms are turned into spectral features through FFT-based power estimation in predefined frequency bands. This feature vector is the input to a support vector machine (SVM) classifier. The AR-SVM approach **(right)** concatenates feature vectors from previous epochs (we use 10 epochs) to provide the classifier with a larger receptive field, i.e., broader access to information from the preceding neural dynamics.

This first feature-based method, adapted from He et al. (2021), computes a periodogram from epoched multichannel waveforms to estimate power in the following frequency bands: θ (2–7 Hz), α (8 – 12 Hz), β_*low*_ (13 – 20 Hz), β_*high*_ (21–30 Hz), γ_1_ (31–45 Hz), γ_2_ (46 – 55 Hz), γ_3_ (56 – 75 Hz), γ_4_ (76 – 95 Hz), γ_5_ (96 – 105 Hz), γ_6_ (106 – 145 Hz), γ_7_ (146 – 155 Hz), γ_8_ (156 – 195 Hz). Each of these features is z-scored with mean and standard deviation values computed from the training feature set, and a support vector machine (SVM) with radial basis functions is trained on the two-class classification problem (see Figure 8). An inner cross-validation loop is used to determine the optimal degree of regularization.

#### 2.3.2. Feature-based model II: autoregressive spectral SVM (AR-SVM)

This system operates with the same feature set and classifier specification as the Spectral SVM, but features are concatenated from the preceding 10 epochs, giving the classifier access to the dynamics from the preceding 4 s as opposed to solely the last 500 ms, thereby increasing its receptive field (Figure 8).

#### 2.3.3. Deep learning-based model I: 1D-CNN

With this model, the multichannel waveforms are sequentially processed through 7 layers. Each layer consists of a custom-made non-linear compression operation, a 1D convolutional layer with a 35-element kernel, a Swish nonlinearity (Ramachandran et al., 2017), an average pooling layer with kernel size 3, and a batch normalization layer (Ioffe and Szegedy, 2015). After the sequence of convolutional layers, an adaptive mean pooling layer is used to reduce each convolutional output to a single scalar value (i.e., feature), which is in turn fed to a linear layer that maps a linear combination of the features onto a final logit.

#### 2.3.4. Deep learning-based model II: autoregressive 1D-CNN (AR-1D-CNN)

**FIGURE 9:**
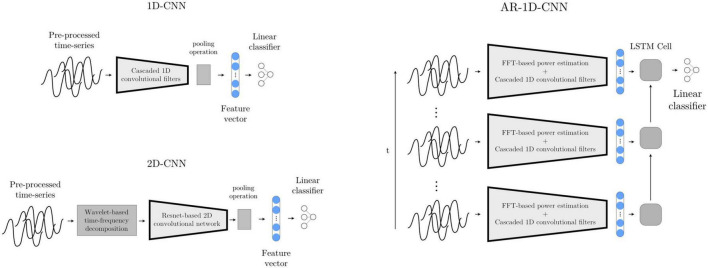
Deep learning-based models 1D-CNN, 2D-CNN, and AR-1D-CNN. When using the 1D-CNN approach **(top left)**, the pre-processed multichannel neural time-series are processed by a filterbank of convolutional filters interleaved by non-linearities prior to pooling and a linear projection onto a logit. The 2D-CNN **(bottom left)** works similarly, but the multichannel input time-series are time-frequency decomposed into 2D-signals that resemble images using wavelets. The network consists of 2D kernels that operate simultaneously in the time and frequency dimensions. The AR-1D-CNN **(right)** works similarly to the 1D-CNN in terms of the way in which it treats individual epochs, but this convolutional feature extraction process is encapsulated within an autoregressive model. This autoregressive model learns relationships contained within sequences of feature vectors. At inference time, the current model output depends not only on the features extracted from the current epoch, but also from the information extracted from previous epochs. The receptive field of the model in the time dimension is thereby augmented. The classifier in this model is given access to, besides the features extracted by the convolutional layers, the spectral features that are manually extracted.

In order to also give our deep learning-based classification systems access to a longer time history (analogously to the AR- SVM), we implement a deep learning-based autoregressive system that has access to the same time course as the AR-SVM. We further augment this system by not only providing it with the features extracted by the learned convolutional filters, but also with the manually extracted features used by the feature-based implementations. To achieve this, the scalar outputs of the 1D-convolutional network (as introduced above) are concatenated with the spectral features used by the SVMs, and this extended feature set is then fed into a recurrent neural network (LSTM with 2 hidden neurons). The output of the last LSTM cell, which incorporates the relevant time-course information, is fed into a linear layer that computes the final logits (Figure 9). At inference time, this system works recursively, i.e., with the LSTM layer keeping track of an internal state within which the relevant historical information is stored and updated. As each new epoch of data is passed through the network, the historical data encoded in the LSTM state influences the current prediction, and this internal LSTM state is updated.

#### 2.3.5. Deep learning-based model III: 2D-CNN

Most well-known convolutional networks were developed for computer vision applications, and thus take in 2D signals (i.e., images) as inputs. To use these architectures with 1D signals, it is necessary to make use of mappings that project 1D waveforms to 2D space. This can be achieved through timeseries encoding techniques such as time-frequency decompositions or Gramian angular fields (Wang and Oates, 2015). Here, we use a wavelet-based time-frequency decomposition to extract instantaneous spectral power estimates between 8 and 200 Hz. The number of cycles of the Morlet wavelets are set to one sixth of the wavelet frequency in Hz (e.g., a 42 Hz wavelet will have 7 cycles). These time-frequency decomposed signals, now with two dimensions per channel, are then fed into a ResNet-based convolutional neural network (please see code implementation for further architectural details) (Figure 9).

The deep learning systems, initialized with random weights for each fold, are trained to minimize the binary cross-entropy loss between the model estimates and the true targets using an Adam optimizer with a learning rate of 0.001 that is annealed every 50 epochs by a multiplicative factor of 0.1, and weight decay (*l_2_*-regularization) of 1e-5. Training runs for a total of 150 epochs, with the model parameters being checkpointed at the end of every epoch and, at the end of training, the checkpoint with the best validation performance is chosen as the final state. Data augmentation is implemented in two ways: once in logit-space by using manifold mixup (Zhang et al., 2018), and also directly on the input waveforms in the form of random additive 1/f-noise with an RMS power drawn from the distribution 𝒰[0.0, 0.2] ⋅ *rms*(*signal*).

The performance metric used to evaluate all models is the balanced accuracy score (Brodersen et al., 2010), which is defined as the arithmetic mean of sensitivity and specificity, and, in terms of the number of samples found in each quadrant of a confusion matrix (TP = # true positives, TN = # true negatives, FP = # false positives, FN = # false negatives) can be described as


$$b\,a\,l\,a\,n\,c\,e\,d_{-}\,a\,c\,c\,u\,r\,a\,c\,y=\frac{1}{2}\,\left(\frac{T\,P}{T\,P+F\,N}+\frac{T\,N}{T\,N+F\,P}\right)$$

### 2.4. Data and code availability

All code (written in python) used to generate the data, train the models and generate the figures is available at https://github.com/fer-rplazas/feature-extraction-methods, and the data is available at https://data.mrc.ox.ac.uk/lfp-et-dbs (DOI:10.5287/bodleian:ZVNyvrw7).

## 3. Results

### 3.1. Dataset I: synthetic data

**FIGURE 10:**
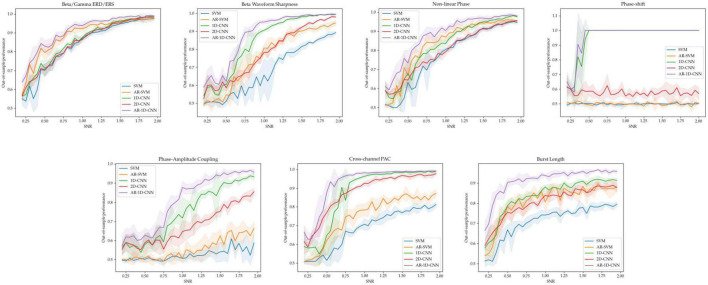
Out-of-sample performance for all 5 models across tasks and SNRs. For each of the 7 tasks, the average performance of each model and the standard deviation (shaded region) across 5 independent runs is shown at each SNR level.

For each of the 7 oscillatory patterns, we generated 5 datasets at each of 36 signal-to-noise ratios ranging from 0.2 to 2. This results in 180 datasets per oscillatory pattern. The out-of-sample performances (measured using the balanced accuracy score) for these SNR sweeps are depicted in Figure 10.

As expected, higher SNRs generally result in better decoding performance. When the different states are distinct in terms of the relative power of oscillators in the beta and gamma bands, all 3 non-autoregressive models perform similarly well. Increasing the receptive fields of models by providing them with access to a longer time-course through autoregressive modeling also increases performance. Importantly, the 1D-CNN-based approaches, which take raw waveform data as input (rather than processed features), perform equally well as the feature-based methods – which explicitly use estimates of power in the beta and gamma bands as inputs. This suggests that, even under noisy conditions and with limited quantity of training data, the 1D-CNNs can make use of the features in the frequency domain for decoding.

For the states distinguished by the sharpness of the beta waveform, the 1D-CNN models perform better (both in their autoregressive and non-autoregressive form) than their feature-based counterparts. The 2D-CNN’s performance here lies in the middle. For the non-linear phase case, the models based on 1D-CNNs slightly outperform their feature-based counterparts, with autoregressive models again boosting performance in both cases. The 2D-CNN performs in line with the base, non-autoregressive SVM. For distinguishing states characterized by features that are not explicitly estimated in the feature set used by the SVMs, including degree of phase shift, phase amplitude coupling (PAC), and burst duration, the CNN-based models perform better than feature-based methods. For distinguishing between degrees of phase-shift, the 1D-CNNs are able to achieve 100% performance starting at a SNR of approximately 0.5, while the feature-based methods and the 2D-CNN perform at around chance level, failing to learn any meaningful relationships between inputs and labels. For distinguishing between PAC levels, all CNNs (including the 2D version) perform better than feature-based methods. This difference is larger when PAC occurs within a single channel than across channels.

To distinguish long and short bursts, the models based on CNNs perform better than the feature-based methods. As in all cases, autoregressivity yields an increase in out-of-sample performance also here, with the AR-SVM being, in terms of classification performance, approximately in line with the non-autoregressive 1D and 2D-CNNs.

Average model performances across the range of observed SNRs can be found in Table 1. Paired t-statistics across model performances allow us to quantify the performance differential in a pairwise fashion, avoiding the need to use mean values over parameter ranges (such as the SNR). In the table below we report on the t-statistics compared with the spectral SVM model. Overall, AR-1D-CNN provided the best decoding accuracy for all scenarios we simulated, this was followed by the 1D-CNN and then the 2D-CNN.

**TABLE 1:** Average model performance (balanced accuracy score) across SNRs.

Pattern (mean balanced accuracy ± mean of std. deviations at each SNR level)	SVM	AR-SVM	1D-CNN	2D-CNN	AR-1D-CNN
Beta/Gamma ERD/ERS	0.84 ± 0.020	0.89 ± 0.018	0.86 ± 0.017	0.85 ± 0.016	**0.91** ±**0.017**
Beta waveform sharpness	0.68 ± 0.032	0.76 ± 0.034	0.84 ± 0.026	0.78 ± 0.018	**0.87** ±**0.019**
Non-linear phase	0.78 ± 0.022	0.84 ± 0.026	0.84 ± 0.022	0.80 ± 0.018	**0.88** ±**0.022**
Phase-amplitude coupling (PAC)	0.52 ± 0.026	0.54 ± 0.036	0.73 ± 0.043	0.66 ± 0.035	**0.79** ±**0.033**
Cross-channel PAC	0.68 ± 0.026	0.75 ± 0.034	0.87 ± 0.035	0.88 ± 0.018	**0.93** ±**0.021**
Phase-shift	0.50 ± 0.01	0.50 ± 0.016	**0.96** ±**0.024**	0.58 ± 0.038	**0.96** ±**0.016**
Burst length	0.71 ± 0.025	0.81 ± 0.036	0.84 ± 0.021	0.81 ± 0.024	**0.91** ±**0.019**
Average cross-pattern performance (paired *t*-test vs. SVM)	0.66 ± 0.023 (n/a)	0.72 ± 0.028 (34.55)	0.84 ± 0.027 (41.42)	0.76 ± 0.024 (44.72)	**0.89** ± **0.021 (56.64)**

Bold values represent highest average performance.

### 3.2. Dataset II: LFP-based movement decoding

**FIGURE 11:**
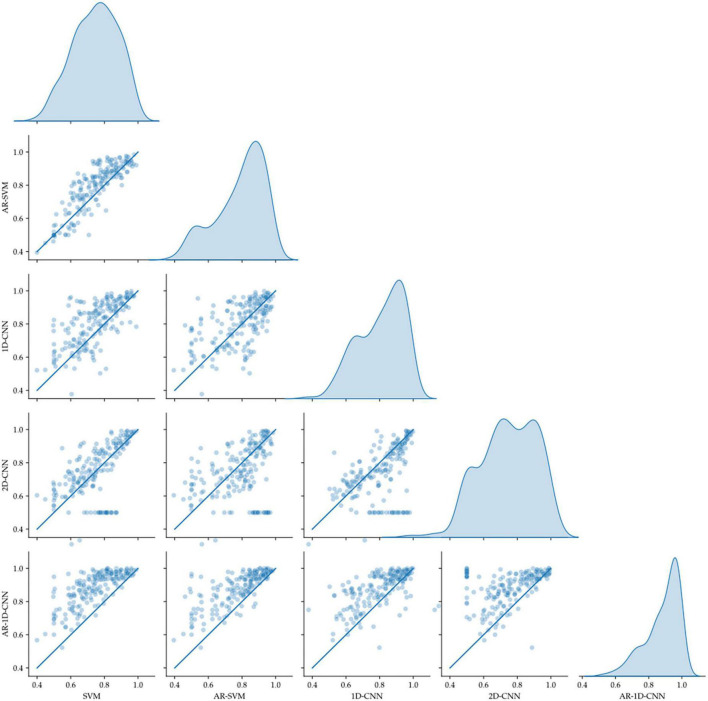
Pairwise model comparison for individual fold (scatter plots, line indicates equal performance), together with the distribution of performances (right-most column). The AR-1D-CNN distribution is most skewed toward higher performance.

We trained 40 datasets recorded from 8 participants with each of them performing 2 to 3 different tasks. Utilizing a 5-fold cross-validation strategy, this results in a total of 200 folds. The pairwise out-of-sample performance for each model pair at each fold is depicted in Figure 11.

Average model performances on an individual fold basis (and corresponding paired *t*-tests against the spectral SVM, which we utilize as a baseline model against which to compare the others) and for fold-averages are outlined in Table 2. We observe that performance metrics at the individual fold level as well as at the fold-average level yield equivalent results.

**TABLE 2:** Average model performances.

	SVM	AR-SVM	1D-CNN	2D-CNN	AR-1D-CNN
Model performance using individual folds: mean ± std. deviation (paired *t*-test vs. SVM)	0.74 ± 0.13 (n/a)	0.79 ± 0.14 (*t* = 8.13, *p* < 1e−10)	0.80 ± 0.13 (*t* = 8.33, *p* < 1e−10)	0.78 ± 0.16 (*t* = 8.24, *p* < 1e−10)	**0.87**± **0.10** **(*t* = 16.7, *p* < 1e**−**10)**
Cross-fold metrics (mean ± std) for each model (paired *t*-test for cross-fold average vs. SVM)	0.75 ± 0.064 (n/a)	0.79 ± 0.08 (*t* = 4.96, *p* < 1.38e−5)	0.81 ± 0.062 (*t* = 5.46, *p* < 2.85e−6)	0.79 ± 0.054 (*t* = 5.42, *p* < 3.3e−6)	**0.87 0.065 (*t* = 11.17, *p* < 1e**−**13)**

Bold values represent highest average model performance.

The AR-1D-CNN, with an average balanced accuracy of 0.87 (for individual folds), performs best out of the 5 models, while the baseline spectral SVM yields the poorest performance (average balanced accuracy of 0.74). The AR-SVM, 1D-CNN and 2D-CNN perform similarly well in terms of average performance (0.80, 0.79, and 0.78, respectively) and utilizing a per-fold, pairwise comparison the 1D-CNN slightly outperforms both the AR-SVM (*t* = 2.24, *p* = 0.026) and the 2D-CNN (*t* = 2.89, *p* = 0.004). Consistent with the results seen in the synthetic data, autoregressive models outperform their non-autoregressive counterparts, and the end-to-end deep learning-based models outperform the feature-based models when the enhanced receptive field due to the autoregressive modeling is accounted for.

## 4. Discussion

### 4.1. Understanding performance differences between the feature-based and deep learning-based approaches

For adaptive DBS and other BCI systems, it could be tempting to try to ascertain whether there is an absolute advantage to either the feature-based or deep learning-based approach—i.e., whether one approach is superior to the other. We, however, do not advocate this view. This is despite the fact that, for all of our experiments, the AR-1D-CNN outperforms all other models. We rather consider that differentials in performance can be explained by concrete factors, such as the individual model’s ability to capture, in its feature space, the signal characteristics that are relevant for a given task. In this respect, both the feature-based and deep learning-based approaches come with respective advantages and drawbacks. These should be taken into account when designing BCI or adaptive DBS systems.

To illustrate this, we point to the performance differentials between feature-based and convolution-based models attempting to distinguish between degrees of *phase shift* and of *beta/gamma power changes* (two of the synthetic signals studied in this work):

• Phase shift: the spectral SVM, by estimating power in canonical frequency bands using a periodogram, discards all phase-related information. It is thus reasonable to expect that, since the extracted feature space doesn’t encode any information related to phase, the performance of these models won’t surpass chance-level if the modulatory pattern of interest is purely phase-related. The 1D-CNNs, by contrast, implements a cascade of FIR filters that are capable of encoding and modeling phase information, and thus perform very well on this task.
• Beta/gamma power changes: For this task, all of the relevant activity takes place in two frequency bands that are captured well by the manual feature extraction process (namely beta and gamma). In such cases, when the information related to the states of interest is captured well in the feature-space that the classifiers have access to, there is no additional benefit to using deep learning methods that are computationally more costly.

In terms of the manually extracted feature set used by the spectral SVM, the two waveform patterns mentioned above correspond to two extremes of the feature space’s ability to model the signal characteristics of interest. In one of the cases, changes in the relevant signal characteristics do not cause any change in the spectral features, and the classifier thus remains agnostic about this change. In the other case, changes to the relevant signal characteristics are entirely reflected in feature space, so that the classifier can easily capture this information. Other scenarios including waveform sharpness, non-linear phase, phase-amplitude coupling and burst length lie somewhere along this spectrum. These features will also lead to changes in the power spectra and will therefore be partially captured by linear transformation methods such as the FFT (see Figures 3–7), even if the predetermined frequency bands have not been specifically tailored to capture these signal characteristics. In these cases, the spectral SVM shows a limited ability to capture the states of interest, but the convolution-based systems, due to their ability to extract arbitrary features from timeseries with fewer constraints, will tend to outperform.

We furthermore point out that dichotomous thinking about feature-based and deep learning-based systems can be misleading in the following way: once a convolutional system has been trained, the learned features can be used (computed through convolutions or otherwise approximated) as part of a feature-based method that uses a SVM as a classification system, and this should yield equivalent results. This has the implication that, when a performance differential between feature-based and deep learning-based methods is observed, a feature set that would bring the feature-based performance up to par with the deep learning-based methods *always* exists – although this does not negate the difficulty of finding and efficiently constructing such a feature space. The relevant consideration is therefore, in our view, not the machine learning approach that one chooses (whether feature-based or deep learning-based), but the design of the features space. Deep learning-based methods (sometimes called feature or representation learning) should thus be thought of as automated feature-learning procedures—this is sometimes referred to as representation learning (Goodfellow et al., 2016).

In this work, we are interested in studying the differences between manually extracted feature sets and learned feature sets extracted through convolutions. We were able to show that CNNs are capable of making use of various features that have been previously observed in neural activities. In our implementation of the AR-1D-CNN, we provide the classifier with access to both the manually extracted spectral features, and the convolutional features learned during the training process. This raises the question of which feature set is more relevant to perform the classification, and investigating this could provide insights into the relative usefulness of the two feature sets.

We investigate this by, first, training the same network on the same data twice, once with and once without the manually extracted spectral features. We then take networks that have been trained *with* the spectral features and upregulate and downregulate the activations that correspond to spectral and convolutional features, while we measure the response in the subsequent activations within the network. Using this procedure, we ascertain whether the network has learned to preferentially “attend” to either manually extracted features or learned convolutional features, and which feature set contributes more to the decision-making process within the network.

**FIGURE 12:**
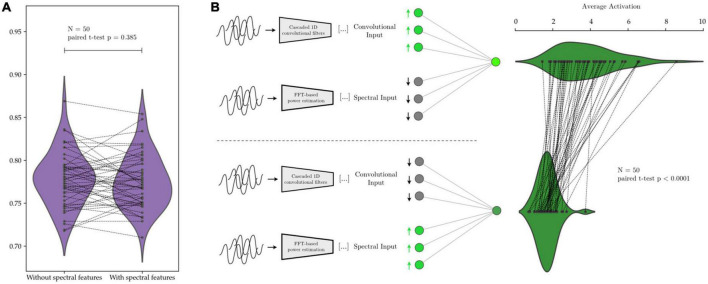
Relative contribution to decoding performance of features extracted by the convolutional network’s layers vs. of predefined features extracted manually. In panel **(A)**, the same network is trained on the same data with and without the predefined spectral features. The average performance is equivalent in both scenarios (paired *t*-test *p* = 0.385). In panel **(B)**, after network training, the inputs corresponding to manually extracted spectral features cause, upon upregulation, a smaller response in network activation than the upregulation of inputs corresponding to features extracted by the convolutional layers of the network. This shows that the network assigns higher weight to the information contained within the learned convolutional features (activation difference paired *t*-test *p* < 0.0001).

We illustrate this in Figure 12 with AR-1D-CNN models that have been trained to distinguish between states of *short vs. long beta bursts* in synthetically generated data. No degradation in performance is observed when the model is not given access to the manually extracted features (Figure 12), indicating that access to the manual feature set it not necessary for the AR-1D-CNN to solve this task, nor to increase performance. Activations in response to an upregulation of convolutional feature inputs are also higher than in response to an upregulation of manually extracted features, indicating that the network has learned to weigh the convolutional features higher in terms of contribution to network output.

In practice, our findings from this study have the following implications: for tasks in which a good understanding of the modulatory patterns of interest is available (and if these patterns can be quantified well in real time), manually extracting features and combining them with simple machine learning methods should provide adequate performance. In these scenarios, it is unlikely for there to be substantial benefit to using deep learning-based methods. This is in contrast with tasks in which the modulatory patterns of interest are not well known. In such cases, and as we have shown in this study, deep learning-based methods can make use of different idiosyncratic features contained within the oscillatory activities to yield performance increases.

In brief, deep learning-based methods excel at feature discovery. They do not yield any additional benefits when the features of interest are known and can be appropriately captured by a manually designed feature-extraction process. However, when the patterns within the data are unknown or not readily quantifiable, or there are too many potential features which might contribute to decoding, thereby making it challenging to extract all potential features, deep learning-based methods may outperform feature-based methods.

### 4.2. Exploring the learned representations within convolutional layers

In this project, we have reported that convolutional neural networks can extract features that have previously been shown to be important in electrophysiology, such as power increase in specific frequency bands, phase shifts or phase amplitude coupling in different neural oscillations. We also reported that features learned by convolutional neural networks can lead to higher decoding performance when compared to a baseline method that uses a rudimentary set of manually extracted spectral features. We have, however, not reported on the potentially novel features that convolutions can learn from data to enable the observed increase in decoding performance shown in Figure 11 and Table 2. This question is relevant for two reasons. First and foremost, because of the necessity to understand the signal characteristics that autonomous systems depend on and react to in the context of BCI systems, adaptive DBS, and clinical neurosciences more broadly. Without this knowledge, it becomes difficult to understand and prevent the failure modes of these autonomous systems as well as the robustness and risks that these systems pose in real-world settings. Secondly, understanding the features learned by learning algorithms might offer new insights into the functional physiology and the pathophysiology of neural circuits in healthy and diseased states, providing a novel and valuable data-driven research tool.

In spite of this, we find a distinct dearth of well-established methodological tools to systematically address this question. This makes us, in agreement with other researchers in the field of electrophysiology (Merk et al., 2022a), at this stage consider neural networks to be systems that, despite their usefulness, have poor interpretability. This is in spite of our knowledge that much research has been conducted to alleviate this, particularly in the field of computer vision (Chattopadhyay et al., 2018; Desai and Ramaswamy, 2020; Selvaraju et al., 2020; Wang et al., 2020).

**FIGURE 13:**
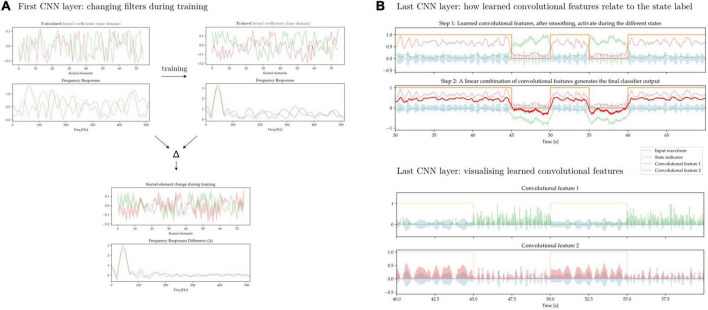
1D-CNN filters and activations. **(A)** Two convolutional filters, along with their frequency responses, before and after network training (top). Change in individual kernel elements as well as frequency responses after training (bottom). During training, these early filters are modified to increase their response to oscillatory activity taking place at lower frequencies. This is expected if the relevant activity is taking place in the beta band. **(B)** Output of the CNN after the last convolutional layer prior to classification. Through a sequence of convolutional operations with filters akin to those seen in panel **(A)** interleaved by non-linearities, two features have been learned (green and pink lines). These two learned convolutional activate preferentially during the two different states, and a linear combination of them yields the ultimate classifier output (red line, top). The learned features do not exhibit linear behavior and are activated by states distinguished by an underlying signal characteristic that is also non-linear (bottom).

In Figure 13 we aim to illustrate some of the difficulty of interpreting the features learned by convolutional neural networks. We first train a 1D-CNN to distinguish between periods of synthetically generated *short vs. long beta bursts.* Figure 13A shows the individual kernel elements, as well as the corresponding frequency responses (1D-CNN kernels can be interpreted as FIR filters), before and after training. Figure 13B shows two learned convolutional features, and how a linear combination of them approximates the ground-truth label. The features, in spite of being composed of linear convolutional filters, exhibit non-linear behavior due to the interleaved non-linear Swish operations.

### 4.3. Choosing time-windows and autoregressive parameters

When training classification systems, a relevant hyperparameter to consider is the length of each epoch. Longer epochs allow for more stable feature estimation due to decreased statistical noise, but come at the cost of decreasing the system’s output rate as well introducing of inertia, thereby rendering the classifier’s response to changes in the underlying signals slower. A target output rate (e.g., 10 classification outputs per seconds, or 10 Hz classification rate) can be maintained by overlapping epochs. Unwanted inertia or reduced decoding accuracy may still be introduced if the epochs are longer than the brain state of interest, even if the epochs are overlapped.

In this work, the autoregressive models introduce a third hyperparameter: the sequence length, i.e., the number of trailing epochs that are used to, at each classification sample in the training data, train the AR-SVM and AR-1D-CNN. When comparing autoregressive and non-autoregressive models, these parameters can also be thought of as determining whether a single epoch is best subdivided into frames, with each frame treated as an element in a sequence within an autoregressive schema, or whether epochs are best kept as a single frame without any subdivisions.

**FIGURE 14:**
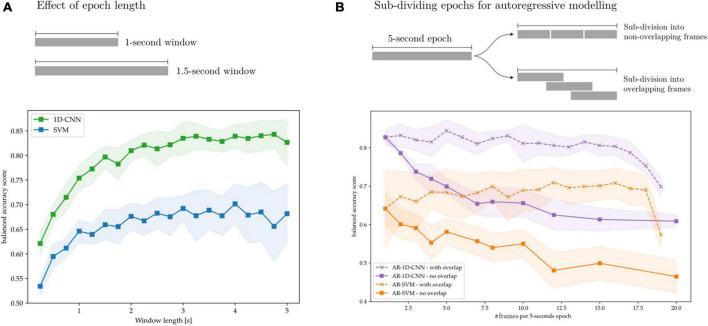
Effect of time windows on decoding performance. **(A)** Overlapped, long time-windows allow for more stable feature estimation, increasing decoding performance without compromising classifier output rate. **(B)** 5 s epochs are subdivided into overlapping and non-overlapping frames that are treated as sequence elements in autoregressive models. Without frame overlap, subdivision of long frames degrades performance, which might be attributable to decreased feature stability due to the shorter feature computation epochs. By overlapping the frames this performance degradation can be avoided.

Here we explore the effect of these design parameters on decoding performance. To this end, we train SVMs, 1D-CNNs, AR-SVMs, and AR-1D-CNNs on the task of distinguishing between periods of synthetically generated short vs. long beta bursts. In Figure 14A, we illustrate that longer time-windows are associated with improved decoding performance for both the SVM and 1D-CNN. We attribute this to more stable feature estimation due to reduced noise and improved sampling to compute each estimate. In our experiments, performance degradation is particularly strong in the sub-500 ms range. This might be attributable to the fact that these very short time windows cannot effectively capture the bursting dynamics of oscillations, which requires having access to enough oscillation cycles to constitute a full long burst (each oscillation at a beta frequency of 20 Hz is 50 ms long).

In Figure 14B, we show the effect on decoding performance of subdividing a 5-s epoch into frames for the AR-SVM and AR-1D-CNN. We use two subdivision strategies: overlapping and non-overlapping frames. When using non-overlapping frames, performance degrades rapidly with subdivisions for both the CNN and SVM. This indicates, analogously to the results shown in Figure 14A, that the model displays a preference for longer time windows. Furthermore, this shows that autoregressive schemas are not able to seamlessly fuse the information contained in feature vectors from two adjacent frames into the equivalent feature vector that would be extracted from the single, unitary frame. This holds for both the AR-SVM and AR-1D-CNN. We attribute this, again here, to improved stability of feature estimation if the frame used to compute each feature is longer. Further research is needed to develop a more thorough understanding of the capabilities of autoregressive models in this context and their relationship with the stability of features. When adjacent frames are overlapped, this allows for longer individual frames from which to extract features and, correspondingly, more stable feature estimation. With this strategy, the performance degradation associated to sub-dividing epochs into frames can be avoided. In our experiments we, however, do not observe any performance benefits to sub-division strategies, whether with overlapping or without, when compared to using whole epochs.

Although we here show the benefits of using longer time windows experimentally and attribute this improvement in performance to more stable feature sets, we do not show the detrimental effect of using epochs that are too long. The synthetic data used here, modeled as a first-order Markov process, has stochastic yet stable state dynamics that are known, with each stable state lasting 10.92 +/- 7.94 s. This might not be the case in arbitrary real-world data. If the changes in underlying brain state dynamics are complex (Vidaurre et al., 2017), long epochs might average out relevant state transitions. The balance between sufficiently long time windows to enable stable feature estimation, but short enough to allow to model state transitions in the neural activities at the appropriate time scale, must be explored on a case-by-case basis.

### 4.4. Inference-time computational cost

**FIGURE 15:**
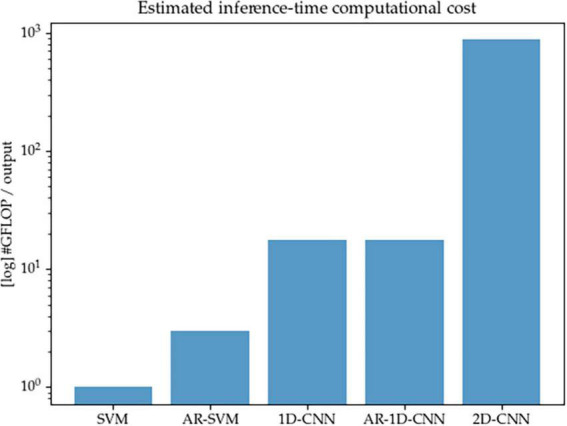
Estimated inference-time computational cost (in number of floating point operations per decoding output, log scale) for each of the methods implemented in this paper. The 2D-CNN, due to its wavelet-based time-frequency decomposition prior to entering the network, and the required network depth in order to achieve sufficient receptive field due to the small kernels used by computer vision models, is the most expensive.

We acknowledge that one key consideration for the real-world implementation of aDBS systems is that the proposed algorithms must remain compatible with the constraints imposed by implantable hardware, such as limited computational resources and restricted power consumption. This can pose a challenge for deep learning (DL) methods, which consist of layers of parametrized matrix operations that well-known to be intensive in terms of compute—and the models described here are no exception. In this work we set out to better understand and compare the modeling capabilities of different methods, rather than to optimize performance-to-compute ratios. For the purposes of the work described here, we don’t directly design systems that operate at very low computational cost. With the current configuration of the models, as shown in Figure 15, 1D-CNNs require approximately one order or magnitude higher of inference-time compute than their SVM counterparts (given the fixed number of features we considered here), and the 2D-CNN in turn requires another order of magnitude above the 1D-CNNs. This is due to both the initial wavelet-based time-frequency decomposition, as well as the short kernels used in 2D convolutional networks that are required to achieve a sufficiently large receptive field.

In future work, the trade-offs between compute and performance should be explored and the question of reconciling the computational requirements of well-performing algorithms with the limited computational resources available in an aDBS context should be explicitly addressed. We point out that model compression methods, including model distillation or the use of streamlined architectures, are able to reduce the inference-time computational footprint of models while minimally compromising on decoding performance. In this study, we have made no attempt to reduce the size of the models or to explore compute/performance trade-offs, but point to this as a worthwhile avenue of research to explore.

### 4.5. Limitations and future work

Once a CNN is trained, it is not trivial to understand the precise computation that it is performing to map input (timeseries signals, in the case of our 1D-CNNs) to output (labels). Although numerous so-called “feature attribution techniques” have been developed for CNNs, mostly in the context of computer vision (2D-CNNs), the fact that these feature-attribution procedures are not designed to be applied systematically across a whole dataset, and furthermore provide insights that are non-trivial to interpret, makes many researchers still consider CNNs to be, at least in part, “black-box” algorithms. These attribution methods are what is generally referred to as “investigating (or visualizing) the activations with CNN layers.” But these tools don’t yet offer a systematic approach to understand features learned by convolutional architectures, especially across a time-series dataset. We view this lack of insight as a current limitation of using CNNs in research and clinical practice. For the type of research that the machine learning community is interested in pursuing, where the existence of a mapping function *f*_*actual*_ : *input* → *output* is assumed to exist, and the role of the algorithm is simply to find the technique that minimizes the error E{*f*_*actual*_ (*input*), *f*_*approx*_ (*input*)}, (where *approx*. refers to the mapping function learned by the SVM, CNN, or algorithm in question), this might be appropriate. Within this machine learning paradigm, developing understanding about how different algorithms process information internally comes only secondary to improving performance. We do, however, not advocate this view in neuroscience, and especially in translational neuroscience, where thoroughly understanding the signals that our systems rely on to operate becomes crucial to ensure the robustness and generalization of these systems beyond controlled clinical settings. Further research is needed to develop the methodological tools that will enable a better understanding of these algorithms’ decision making, and the corresponding failure modes that clinicians and researchers can expect from these systems during real-world use.

## 5. Conclusion

In this work, we explored different decoding methods in terms of their ability to identify states from neural oscillations and benchmarked their performance on both simulated data and LFP data recorded from externalized essential tremor patients. We were able to show that end-to-end convolutional neural networks can make use of different spectral and time-domain features in the oscillatory activities, including power changes, waveform sharpness, phase-amplitude coupling, burst duration, phase shifts, and non-linear phase characteristics. 1D-CNNs trained end-to-end can yield superior performance, especially in cases where the underlying features of interest are not well-known or not easily captured by standardized feature extraction pipelines.

## Data availability statement

The original contributions presented in this study are included in the article/supplementary material, further inquiries can be directed to the corresponding author.

## Ethics statement

The studies involving human participants were reviewed and approved by the South Central – Oxford C Ethics Committee. The patients/participants provided their written informed consent to participate in this study.

## Author contributions

FR wrote the code, performed the signal and statistical analyses, and wrote the manuscript. SH and HT reviewed and revised the project and manuscript. HT organized the project. All authors contributed to the article and approved the submitted version.

## Footnotes

1
https://github.com/neuromodulation/py_neuromodulation

2
https://github.com/felixpatzelt/colorednoise
